# A New Species of *Megastigmus* and First Record of the Genus and Megastigmidae Family from the Paradise of the Maldives Archipelago †

**DOI:** 10.3390/insects14080677

**Published:** 2023-07-31

**Authors:** Irinel Eugen Popescu, Irina Neta Gostin

**Affiliations:** Faculty of Biology, “Alexandru Ioan Cuza” University of Iași, Bdul Carol I, no. 11, 700506 Iasi, Romania; irinagostin@yahoo.com

**Keywords:** Maldives, islands biogeography, *Megastigmus irinae* new species, *Pemphis acidula*, phytophagous, seed eater insect, host plant, SEM

## Abstract

**Simple Summary:**

*Pemphis acidula* is a relative common plant on the seashores of the Maldives islands but it can be found in the coastline and mangroves zones from East Africa to Southeast Asia, Australia, Micronesia, and French Polynesia. We describe a new, very tiny species measuring around 1.6 mm in length, which develops as a larva inside the seeds of *P. acidula*, with one larva per seed. We photographed the live female and male specimens and used advanced microscopy techniques, such as scanning electron microscopy, to provide a detailed description of this new species. This research sheds some light on the Megastigmidae family and *Megastigmus* genus by discovering a surprising new species that provides interesting data on morphology and biology. It was conducted in the paradise of the Maldives Archipelago, offering insights into islands biodiversity and biogeography, climate change, and habitat destruction.

**Abstract:**

Megastigmidae comprises more than 200 species in 12 genera. *Megastigmus* has a worldwide distribution with more than 150 species. Over 80% of these species are recorded from the Australian and Palearctic region, with a few from Afrotropical and Oriental regions, but none from the Neotropical region. We describe a new species of *Megastigmus* obtained from the seeds of *Pemphis acidula* in the Maldives Archipelago. This is the first mention of Megastigmidae having as a host plant a species from Lythraceae. It is also the first recorded association of Chalcidoidea with the genus *Pemphis* and the first mention of Megastigmidae and *Megastigmus* in the Maldives Archipelago. We provide a detailed description of the species, focusing on its morphology, using both light microscopy and scanning electron microscopy (SEM). *Megastigmus irinae* Popescu n. sp. is a strictly phytophagous species, with each larva consuming a single seed. Currently, *M. irinae* is an endemic species found only in the Maldives Archipelago. However, considering the distribution of its host plant, *P. acidula*, which ranges from East Africa to Southeast Asia, Australia, Micronesia, and French Polynesia, we anticipate that future research could significantly expand the known range of this interesting new species.

## 1. Introduction

The superfamily Chalcidoidea Latreille is known for its remarkable diversity, with over 22,000 described species. However, it is believed that the actual number of species within this superfamily far exceeds this estimate, ranging from 100,000 to 500,000 species [1]. Unfortunately, the destruction of habitats poses a significant threat to many species, often resulting in their extinction even before we have the opportunity to discover their existence.

Our understanding of the Chalcidoidea fauna in the Maldives is severely limited, with very little knowledge available on this particular group of insects in the region. Noyes [1] listed four species from two families, three from Aphelinidae: *Encarsia dispersa* Polaszek [2], *Encarsia abundantia* Chou and Su [3], *Encarsia smithi* (Silvestri) [4]; and one from Eulophidae: *Platyplectrus orthocraspedae* Ferrière [5]. There might be some reasons for this. The islands in the Maldives are very small, typically not more than 1–2 km in length and usually less than 1 km wide. The sandy substrate contributes to a relatively low biodiversity on land, with limited trophic niches available. Additionally, the impact of human habitation and tourism further influences the ecosystems. However, in the water, it is an entirely different story! There is still a vast biodiversity, but it has been tragically impacted by global climate change. Currently, some coral atolls resemble vast graveyards, but we must maintain hope for the future. As a scuba diver, the first author is well acquainted with both sides of the underwater biodiversity of the Maldives.

Megastigmidae Thomson was traditionally classified as a subfamily in the Torymidae Walker family. However, Janšta et al. [6] elevated Megastigminae to the family level, and this reclassification is currently accepted [7]. Megastigmidae comprises more than 200 described species in 12 genera [1,7,8], 9 reported from Australia [9]. The Australasian region is considered the center of origin for Megastigmidae [6]. Doğanlar [10] transferred all entomophagous species with a metallic shine from *Megastigmus* Dalman to *Bootanomyia* Girault, while *Megastigmus* now only includes nonmetallic-shine phytophagous seed-feeding species, infesting seeds of Pinaceae, Cupresaceae, Taxodiaceae, Rosaceae, Anacardiaceae, Myrtaceae, and Aquifoliaceae [1]. *Megastigmus* has a worldwide distribution and includes more than 150 described species [1]. Over 80% of the species are recorded from the Australian and Palearctic regions, with a few species from the Afrotropical and Oriental regions, and none from the Neotropical region [11]. 

Genus *Pemphis* J.R. Forst. & G. Forst. (Myrtales: Lythraceae) consists of only two species, *Pemphis acidula* J.R. Forst. & G. Forst. and the endemic *P. madagascariensis* (Baker) found in Madagascar. The native global geographic distribution includes East Africa (Mozambique, Tanzania, Zanzibar, Seychelles), the British Indian Ocean Territory, the Maldives, India, Sri Lanka, China, Taiwan, Southeast Asia (Myanmar, Vietnam, Thailand, Cambodia, Malaysia, Singapore, Indonesia, Papua New Guinea, Palau, the Philippines), Japan, Northwest and Northeast Australia, the Solomon Islands, the Federated States of Micronesia, Fiji, Tuvalu, the Pacific Islands (from the Marshall Islands to Tonga), the Cook Islands, and French Polynesia [12]. 

The Maldives Archipelago is situated in the center of the native global geographic distribution axis of *P. acidula*, which includes East Africa, India, Sri Lanka, and Southeast Asia. *P. acidula* is a perennial shrub that often forms dense brushes along the sandy beaches in the Maldives. Its bark ranges in color from grey to brown, and it has adventitious roots with no special adaptations. The solitary flowers have six white petals, and the fruits (capsules) are surrounded by a persistent calyx. The capsules have a reddish style with circumscissile dehiscence, and the plant produces many angular seeds with a corky margin or wing [13,14]. *Pemphis acidula* is classified as LC (least concern) status in the Global IUCN Red List, primarily due to the global decline of mangrove areas. *P. acidula* is a sturdy and robust plant that is highly beneficial for shoreline protection against high winds, and it can serve as an environmental bioindicator for mangrove conservation [15].

The Maldives Archipelago comprises 26 coral atolls, housing more than 1300 coral islands. It is recognized as the world’s lowest country, with an average natural ground level of 1.5 m and the highest natural point reaching 2.4 m. The total area of the Republic of Maldives spans around 90,000 km^2^, but the dry area above sea level is less than 300 km^2^. Maldives stands as one of the world’s most geographically dispersed countries, with a highly uneven distribution of the human population across various islands. As Domroes [16] eloquently stated, the “Maldives represents a nation of water rather than a nation of islands”, making it particularly vulnerable to becoming a nation of climate refugees due to the rising of the global sea levels, caused by climate change.

The terrestrial fauna of the Maldives is primarily composed of species originating from India, Sri Lanka, Africa, and Seychelles. Some species have naturally migrated to the Maldives, while others have been introduced through human activities [17]. In contrast to the knowledge we have about aquatic fauna, our understanding of the zoogeography of terrestrial insect fauna in the Maldives is still limited [18].

## 2. Materials and Methods

### 2.1. Entomological Material

**Figure 1:**
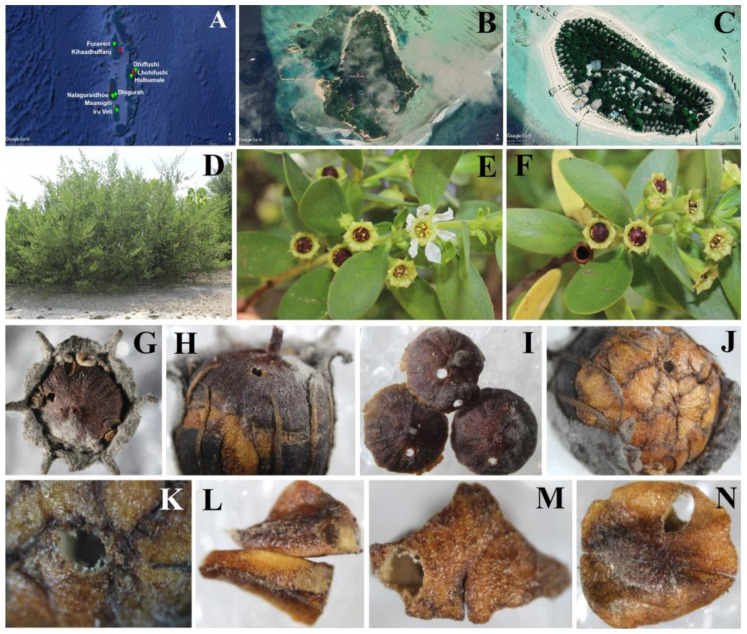
Investigated areas and host plant. (**A**–**C**) Google Earth satellite images; (**A**) the Maldives Archipelago with investigated sites: red—islands with *M. irinae*, green—investigated islands, but *M. irinae* not found; (**B**) Lhohifushi Island; (**C**) Kihaadhuffaru Island; (**D**–**N**) *Pemphis acidula*; (**G**–**K**) capsules with emergence holes of *M. irinae*; (**L**–**N**) seeds with emergence holes of *M. irinae*.

In the period from 2017 to 2022, we conducted expeditions to several islands in the Maldives Archipelago: Nalaguraidhoo (Alif Dhaal Atoll) (2017), Dhigurah (Alif Dhaal Atoll) (2017), Maamigili (Alif Dhaal Atoll) (2017), Dhiffushi (Kaafu Atoll) (2017), Hulhumale (North Male Atoll) (2017, 2020, 2022), Lhohifushi (North Male Atoll) (2018), Furaveri (Raa Atoll) (2020), Iru Veli (Dhaalu Atoll) (2021), and Kihaadhuffaru (Southern Maalhosmadulu Atoll from Baa Atoll) (2022) (Figure 1A). We collected capsules from *Pemphis acidula* at all these locations and used yellow traps and an entomological net for sweeping on vegetations, including on *P. acidula*. We just collected capsules without any hole of emergence. Generally, the expeditions were conducted in July, except in Furaveri (December), Kihaadhuffaru (September), and Hulhumale (once in September and once in December). The Maldives Archipelago is situated close to the Equator and has a tropical monsoon climate that remains constant throughout the year, with a daily mean temperature consistently around 28 °C, an average high of 31.5 °C, and an average low of 26.4 °C. The humidity levels are significantly amplified during the rainy season. Given the very homogenous climate throughout the year, we do not think that the date of collection greatly influenced the results as it might have in other geographical areas.

We collected 128 specimens of *Megastigmus irinae* Popescu n. sp., 104 ♀♀ and 24 ♂♂, 5 ♀♀ and 2 ♂♂ were used for dissections, for microscopic slides, and SEM. In total, 82 specimens, 76 ♀♀ and 6 ♂♂, were collected by sweeping with an entomological net on plants of *Pemphis acidula*, 53 ♀♀ and 6 ♂♂ on Lhohifushi Island, and 23 ♀♀ on Kihaadhuffaru Island. A total of 46 specimens were obtained in laboratory conditions from capsules of *Pemphis acidula* (Figure 1D–K), 9 specimens (3 ♀♀ and 6 ♂♂) from capsules collected on 25.07.2018 from Lhohifushi Island (Figure 1B) and 37 specimens (25 ♀♀ and 12 ♂♂) from capsules collected on 20.09.2022 from Kihaadhuffaru Island (Figure 1C).

### 2.2. Macrophotography

Images of the live female and male specimens were taken using a Canon 100 mm f/2.8 Macro USM lens attached to a Canon 60D digital camera (Canon Inc., Tokyo, Japan).

### 2.3. Light Microscopy

The female holotype and male allotype were photographed using a Leica DFC500 digital camera (Leica Microsystems, Heerbrugg, Switzerland) mounted on a Leica M205A (Leica, Switzerland) automated research stereomicroscope. The images were processed with Zerene Stacker^®^ and their clarity was enhanced using Adobe^®^ Photoshop^®^ CC (20.0.0 Release, Adobe, San Jose, CA, USA).

Body parts were dissected using an Euromex stereomicroscope (Euromex Microscopen BV, Arnhem, Netherlands) with a maximum magnification of 180× and mounted in Canada balsam. Images of the antennae, maxillolabial complex, wings, legs, male genitalia, ovarian eggs, capsules, and seeds of *Pemphis acidula* were captured using a Canon 600D digital camera (Canon Inc.) connected to a ZEISS SteREO Discovery.V20 stereomicroscope (ZEISS Microscopy, Oberkochen, Germany).

Observations and descriptions were conducted using an Euromex stereomicroscope with a maximum magnification of 180×. Measurements were taken using an ocular micrometer. The magnification used for photographing body parts under light microscopy was the same, ensuring that the relative lengths and ratios were comparable.

### 2.4. Scanning Electron Microscopy (SEM)

For morphology micrographs, samples were mounted on stubs with carbon conductive tape, sputter-coated with a 30 nm layer of gold (EMS 550X Sputter Coater) and examined by a scanning electron microscope Tescan Vega II SBH (TESCAN, Brno, Czech Republic), from the Electron Microscopy Laboratory, Faculty of Biology, ‘‘Alexandru Ioan Cuza’’ University of Iași.

### 2.5. Abbreviations Used, Terminology

Collection acronym: IPCO, I. E. Popescu personal collection, University ‘‘Alexandru Ioan Cuza’’, Faculty of Biology, Laboratory of Entomology, Iaşi, Romania. Abbreviations used for morphological characters: HL (head length), HW (head width), HH (head height), BOF (breadth of oral fossa), LMS (length of malar space), LE (length of eyes), HE (height of eye), DAT (distance between antennal toruli), DT (diameter of toruli), LATE (length between antennal toruli and eye), LTOF (length between toruli and oral fossa), LT (length of temples), IOD (inner orbit distance), OOL (ocello-ocular line), POL (posterior ocellar line), LOL (lateral ocellar line), DMO (diameter of median ocellus), DLO (diameter of lateral ocelli), ML (mesosoma length), MW (mesosoma width), PCL (pronotal collar length), PCW (pronotal collar width), MLL (middle lobe of mesoscutum length), MLW (middle lobe of mesoscutum width), SL (scutellum length), SW (scutellum width), FL (frenum length), PL (propodeum length), PW (propodeum width), SMV (submarginal vein), PST (parastigma), MV (marginal vein), PMV (postmarginal vein), STV (stigmal vein), STG (stigma), and UNC (uncus).

The morphological terminology follows Hymenoptera Anatomy Ontology [19], Gibson et al. [20], Grissell [21], and Roques and Skrzypczynska [22]. For the description of a new species from Chalcidoidea we found useful the monography of Gibson and Fusu [23] and for the maxillolabial complex of Hymenoptera, the papers of Popovici et al. [24,25].

The distribution map and satellite images of Lhohifushi and Kihaadhuffaru islands were created using Google Earth Pro^®^ (7.3.6.9345 Release, Google, Santa Clara, CA, USA). The descriptions were based on the holotype female (♀) and allotype male (♂), but we also utilized other specimens for supplementary illustrations, dissections, macrophotography, light microscopy, and scanning electron microscopy (SEM).

## 3. Results

***Megastigmus irinae* Popescu n. sp.**

Figure 2A–I.

### 3.1. Type Material

#### 3.1.1. Holotype ♀ 

**Figure 2:**
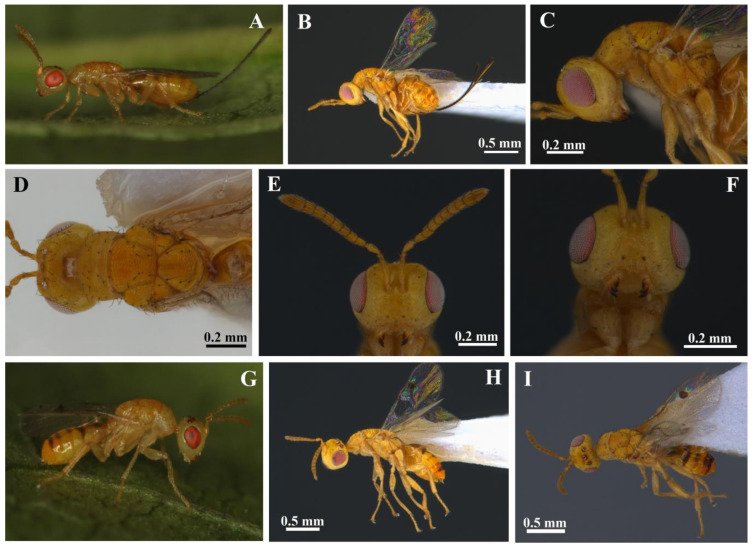
*Megastigmus irinae* ♀♂. (**A**) ♀ live specimen; (**B**–**F**) ♀ holotype; (**G**) ♂ live specimen; (**H**,**I**) ♂ allotype.

Maldives, Lhohifushi Island, North Male Atoll, 4.3498690N, 73.6166750E, 1 m, 24.07.2018, on the seaside, collected by sweeping with an entomological net on plants of *Pemphis acidula* (Lythraceae), I. E. Popescu leg (IPCO). Condition: glued by the right side on the thorax and gaster on the left tip on a triangular card; uncontorted; entire (Figure 2B–F).

#### 3.1.2. Allotype ♂

Maldives, Lhohifushi Island, North Male Atoll, 4.3498690N, 73.6166750E, 1 m, 24.07.2018, on the seaside, collected by sweeping with an entomological net on plants of *Pemphis acidula* (Lythraceae), I. E. Popescu leg (IPCO). Condition: glued by the right side on the thorax and gaster on the left tip on a triangular card; uncontorted; entire (Figure 2H,I).

#### 3.1.3. Paratypes 

Forty-six ♀♀ and twenty-one ♂♂: one ♀, six ♂♂ ex. seeds of *Pemphis acidula*, Maldives, Lhohifushi Island, North Male Atoll, 4.3498690N, 73.6166750E, 1 m, 25.07.2018, I. E. Popescu leg (IPCO). Ten ♀♀, five ♂♂, Maldives, Lhohifushi Island, North Male Atoll, 4.3498690N, 73.6166750E, 1 m, 25.07.2018, on the seaside, collected by sweeping with an entomological net on plants of *Pemphis acidula* (Lythraceae), I. E. Popescu leg (IPCO). Twenty-five ♀♀, ten ♂♂, ex. seeds of *Pemphis acidula*, Maldives, Kihaadhuffaru Island, Southern Maalhosmadulu Atoll from Baa Atoll, 5.1910150N, 73.1334330E, 1 m, 20.09.2022, I. E. Popescu leg (IPCO). Ten ♀♀, Maldives, Kihaadhuffaru Island, Southern Maalhosmadulu Atoll from Baa Atoll, 5.1910150N, 73.1334330E, 1 m, 20.09.2022, on the seaside, collected by sweeping with an entomological net on plants of *Pemphis acidula* (Lythraceae), I. E. Popescu leg (IPCO).

#### 3.1.4. Additional Material in Alcohol 

Fifty-two ♀♀: 39 ♀♀, Maldives, Lhohifushi Island, North Male Atoll, 4.3498690N, 73.6166750E, 1 m) 25.07.2018, on the seaside, collected by sweeping with an entomological net on plants of *Pemphis acidula* (Lythraceae), I. E. Popescu leg, (IPCO). Ten ♀♀ ex. seeds of *Pemphis acidula*, Maldives, Kihaadhuffaru Island, Southern Maalhosmadulu Atoll from Baa Atoll, 5.1910150N, 73.1334330E, 1 m, 20.09.2022, I. E. Popescu leg (IPCO). Thirteen ♀♀, Maldives, Kihaadhuffaru Island, Southern Maalhosmadulu Atoll from Baa Atoll, 5.1910150N, 73.1334330E, 1 m, 20.09.2022, on the seaside, collected by sweeping with an entomological net on plants of *Pemphis acidula* (Lythraceae), I. E. Popescu leg (IPCO).

### 3.2. Description

#### 3.2.1. Holotype ♀

**Figure 3:**
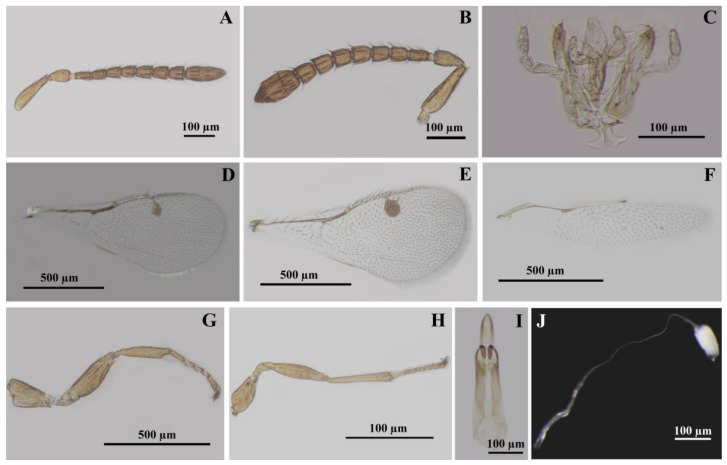
Light microscopy morphology of female and male specimens of *M. irinae*. (**A**) ♀ antenna; (**B**) ♂ antenna; (**C**) ♀ maxillolabial complex; (**D**) ♀ forewing; (**E**) ♂ forewing; (**F**) ♀ hindwing; (**G**) ♀ foreleg; (**H**) ♀ hindleg; (**I**) ♂ genitalia; (**J**) ovarian egg.

**Figure 4:**
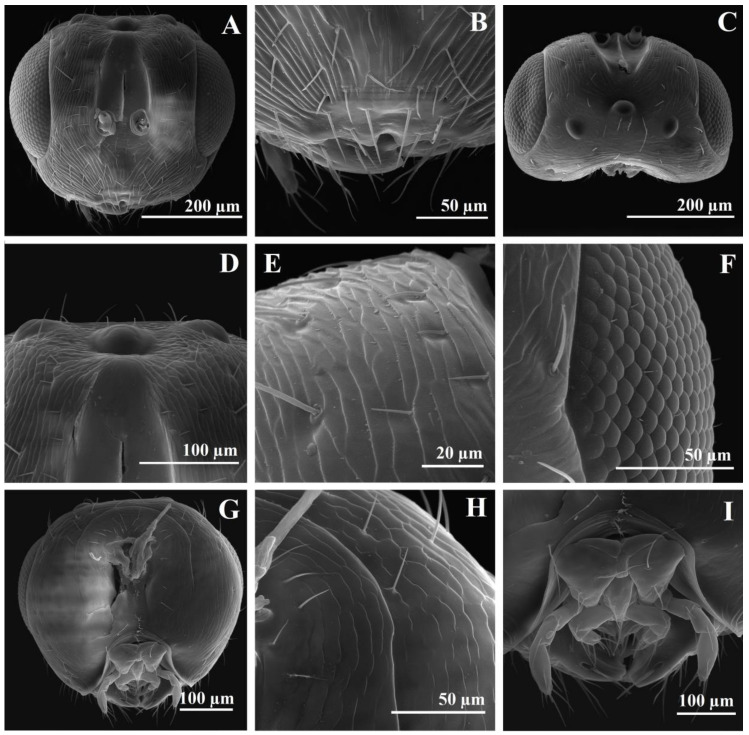
*M. irinae* (♀)—SEM micrographs. (**A**) head—anterior; (**B**) lower face, clypeus; (**C**) head—dorsal view; (**D**) ocelli, scrobal depression, parascrobal areas; (**E**) parascrobal area; (**F**) eye; (**G**) head-posterior view; (**H**) occipital carina; (**I**) maxillo-labial complex.

Body length without ovipositor, 1.60 mm; length of ovipositor sheaths, 1.25 mm. The body color is generally yellow with black scattered setae, with light to paler yellow head, pronotum and legs, yellow to white coxae, especially fore and middle coxae, and some additional amber on the antenna, mesonotum, propodeum, and gaster, with some black on the back of the gaster; the ovipositor sheaths are black (Figure 2A–F and Figure 3A,D,F–H). The head color is light yellow, pale yellow around the eyes, the lower face, scrobal depression, parascrobal area, genae, temple, vertex, and occiput, a darker yellow in the middle of the lower face, supraclypeal area, darker on the frons, with some light amber around the ocelli. The interantennal prominence is light yellow. The scrobal longitudinal carina has a very thin brown to black longitudinal line on the top. The eyes are red, bare on stereomicroscopes but on SEM, they have very scattered minuscule setae (Figure 4F). The ocelli are red. The anterior tentorial pits are brown. The clypeus has a brown terminal on the lobe area. The mandibles are light yellow basally and black distally, especially the dents, with a small brown intergrade area and a brown to black anterior edge. The maxillolabial complex, including the maxillary and labial palps, is pale yellow. The antenna is amber, darker on the pedicel and flagellum; the scape is light amber to yellow in the basal part and the radicle is yellow. All antennomeres have brown sensilla trichodea, all funiculars and all clavomeres have white longitudinal sensilla. The setae are black on the frontovertex, temple, upper face, and genae and white on the lower face, supraclypeal area, clypeus, labrum, and maxillolabial complex, generally with black-dotted setal pores. The pronotum is light yellow to pale yellow up to the suture with the mesoscutum. The lateral panel of the pronotum, propleuron, and prosternum is pale yellow. All setae on the pronotum (pronotal collar) and upper back side of the lateral panel of the pronotum are black, sparse, and hairlike. The middle lobe of the mesoscutum is almost all amber but in dried specimens, it has two visible U-shaped amber areas on the anterior part, separated by a central, thin, light-yellow line. The posterior part of the middle lobe of the mesoscutum is light amber; the lateral areas of the middle lobe to the notauli are light yellow. The lateral lobe of the mesoscutum is amber with light yellow laterally and posteriorly. The scutellum is amber on the central part, inside the area delimited by the six long, black, hairlike setae. The lateral areas of the scutellum, outside the central area delimited by the six setae, and to the suture with the middle lobe of the mesoscutum are light yellow. The frenum is amber anteriorly to light yellow posteriorly. All setae on the mesonotum are long, sparse, black, and hairlike. The axilla is amber with a light yellow to the suture with the scutellum and a thin black basal line. The metanotum is amber to light yellow, especially in the central part (dorsellum) and latero-posterior areas. The propodeum is amber, bare in the central area between the two postspiracular sulci and light yellow on the lateral areas (callus), with dense, short, thin, white setae with black-dotted setal pores. The prepectus is light yellow and bare. The tegula is pale yellow with sparse black hairs. The acropleuron is pale yellow and bare. The mesopleuron is light yellow to pale yellow on the upper part of the upper mesepimeron. The mesepisternum and mesepimeron are bare. The metapleuron on the anterior part, basally, and the anterior face of the mesepisternum has sparse white setae with black-dotted setal pores. The propleuron, prosternum, and mesosternum are light yellow and bare. The prosternal area is black in the center, difficult to see with the fore legs in the anatomical position but easy to see after removing them or lifting the procoxae. The metasternum (metasternal area) is black, difficultly visible with the middle and hindlegs in the anatomical position, easy to see after removing them. The forewings are hyaline with brown setae (Figure 3D). The humeral plate is white. The submarginal vein is white basally to progressively brown in the middle. The parastigma is strongly brown with a light brown to the marginal vein. There are sparse, long, brown setae on the submarginal vein. The marginal vein is white with long brown setae. The postmarginal vein is white. The stigmal vein has a light brown base to the beginning of the stigma; most of the stigma is strongly brown. The uncus is pale brown. The hindwing is hyaline with brown setae (Figure 3F). The humeral plate is white. The submarginal vein is white in the first half to progressively brown in the middle and has a strong brown parastigma. The marginal vein is white with a black spot distally, in the hamuli area. The hamuli are brown. The fore coxa is light yellow, almost white on dried specimens, with short, white, scattered setae with black-dotted setal pores. The fore trochanter is light yellow, almost white on dried specimens. The profemur is light yellow with white, short setae on the interior face, black medium setae on the distal part of the exterior face, and long black setae on the posterior face with black-dotted setal pores on the exterior and posterior faces. The protibia is light yellow with an external longitudinal line of black setae with black-dotted setal pores and short white setae on the other faces. The protibial spur is light yellow. On all legs, the tarsomeres are light yellow, the claws are light brown, the arolium is brown. The mesocoxa is light yellow, almost white on dried specimens, with short, white, scattered setae. The mesotrochanter is light yellow, almost white on dried specimens. The mesofemur and mesotibia are light yellow, both with minuscule white setae on the interior face and small black setae on the exterior face. The mesotibial spurs are light yellow. The hindleg is light yellow. The metacoxa has long, white, scattered setae with black-dotted setal pores on the upper posterior face, medium, white, scattered setae with black-dotted setal pores on the exterior and interior faces. The metafemur has three external longitudinal lines of black setae with black-dotted setal pores. The metatibia has short, black, external setae with black-dotted setal pores and short, white, internal setae. The metatibial spurs are light yellow. The petiole is amber. The gaster is amber dorsally with black transversal wide straps on the second half of gastral tergites 1–6. The lateral sides of the gaster are light yellow. The gastral sternum, including the hypopygium, is light to dark yellow. The gaster is bare, with one transversal subterminal to the median line of the long, hairlike, black setae on gastral tergites 1–6 and few scattered, long, black setae on the distal part of gastral sternum segments. The ovipositor stylet is brown. The ovipositor sheaths are black with black setae. The epipygium is white and the cerci are black, both with white setae.

**Figure 5:**
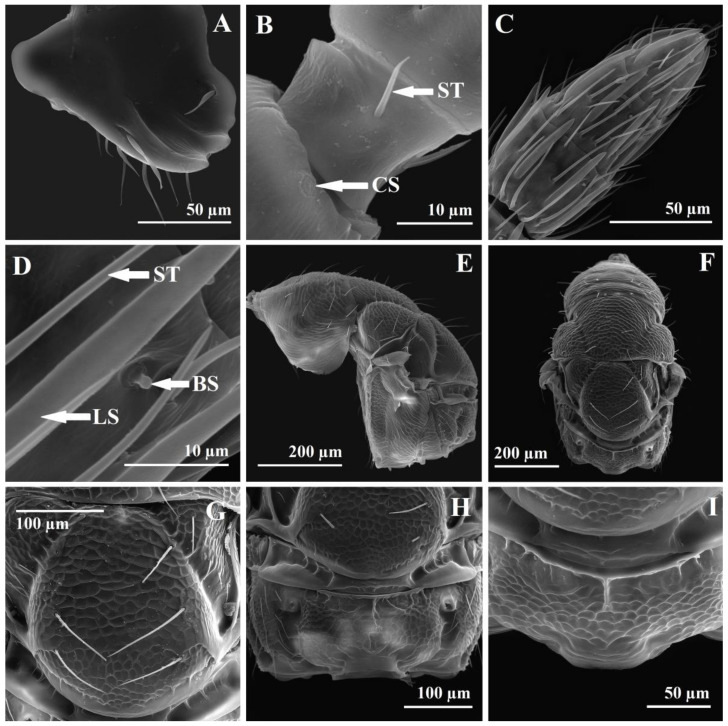
*M. irinae* (♀)—SEM micrographs. (**A**) Right mandible; (**B**) pedicel with campaniform sensilla (CS), anellus with sensilla trichodea (ST); (**C**) clava; (**D**) clava detail with sensilla trichodea (ST), longitudinal sensilla (LS), and basiconic capitate sensilla (BS); (**E**) mesosoma—lateral view; (**F**) mesosoma—dorsal view; (**G**) scutellum; (**H**,**I**) propodeum.

The head is slightly transverse, 1.2× as wide as high and 1.65× as wide as long. The eye is 1.60× as high as long. The length of the malar space is 0.86× as long as the breadth of the oral fossa and 0.72× the eye’s height. The POL is 2× as long as the OOL. Relative measurements of the head: HW 42, HH 35, BOF 15, LMS 13, HE 22, DT 4, DAT 4, LATE 10, LTOF 10, HL 25, LT 4, LE 18, IOD 27, OOL 5, POL 10, LOL 3, DMO 5, DLO 5. The vertex is shallow striate with scattered setae, slightly alutaceous imbricate around the ocelli (Figure 4C,D), frons, and parascrobal areas, more accentuated striate with two long setae in the upper half of the parascrobal areas (Figure 4A). The scrobal depression is smooth with a very thin longitudinal carina (Figure 4A). The lower face is strongly radially striate with scattered short setae (Figure 4A,B). The malar sulcus is very thin. The clypeus is smoothly bilobed (Figure 4B). The mandibles have three dents, bigger basally and in the middle, the upper one very small (Figure 5A) and difficult to see on a stereomicroscope (Figure 2F), especially with the mandibles closed. The mandibles have two sensilla trichodea on the dorsal face (Figure 5A) and seven on the ventral face. The maxillary palps have four articles, and the labial palps have three articles (Figure 3C and Figure 4I). The maxillolabial complex has sensilla trichodea. The stipes have three sensilla trichodea ventrally. The maxillary and labial palps have sensilla trichodea and campaniform sensilla. The occipital carina is very thin, almost impossible to see on a stereomicroscope but well visible on SEM (Figure 4G,H). The hypostomal carina is well developed (Figure 4G). The genae, postgenae, temple, and occiput are shallowly striate to slightly alutaceous imbricate (Figure 4G,H). The vertex to the temple, close to the eye, has a line of five setae. The POL has three setae. The posterior-to-posterior ocellus line has three setae. The postgenal bridge has very thin transversal carinas. The posterior part of the head is almost bare, with few setae on the upper part of the occipital foramen (Figure 4G,H). The antenna is inserted approximatively in the middle of the face (Figure 2E and Figure 4A). The distance between the toruli is equal to the diameter of the toruli and 0.4× as long as the length between the antennal toruli and the eye. The length between the antennal toruli and the eye is equal to the distance between the toruli and oral fossa, the scape not reaching the anterior ocellus. The scape and pedicel are smooth; on SEM, they are very finely longitudinally striate (Figure 5B). The scape is 3.5× as long as broad, the anellus is slightly transverse, the flagellum is 3.28× as long as the scape, the funicle is 2.66× as long as the clava. F1 is slightly longer than the other funiculars, 1.66× as long as broad, and 1.25× as long as F2 (Figure 3A). Relative length/breadth (ratio) of the antennomeres: flagellum 46; funicle 32; scape 14/4 (3.5); pedicel 6/4 (1.5); anellus 1.8/2.1 (0.85); funiculars: F1 5/3 (1.66), F2 4/3 (1.33), F3 4/3 (1.33), F4 4/4 (1), F5 4/4 (1), F6 4/4 (1), F7 4/4 (1); clava: 12/5 (2.4). The clava are without spicula, 3× as long as F7, and 1.25× as broad as F7 (Figure 3A and Figure 5C). All antennomeres have sensilla trichodea, including the radicle and anellus (Figure 3A and Figure 5B–D). The anellus has 2–3 sensilla trichodea (Figure 5B). The pedicel has campaniform sensilla (Figure 5B). All funiculars and all clavomeres have longitudinal sensilla (multiporous plate sensillum, placoidea) (Figure 3A and Figure 5C,D). F1 has 2–3 longitudinal sensilla. The apex of the longitudinal sensilla is longer that the length of the funiculars. Funiculars F6 and F7 and all clavomeres have basiconic capitate sensilla (Figure 5C,D).

**Figure 6:**
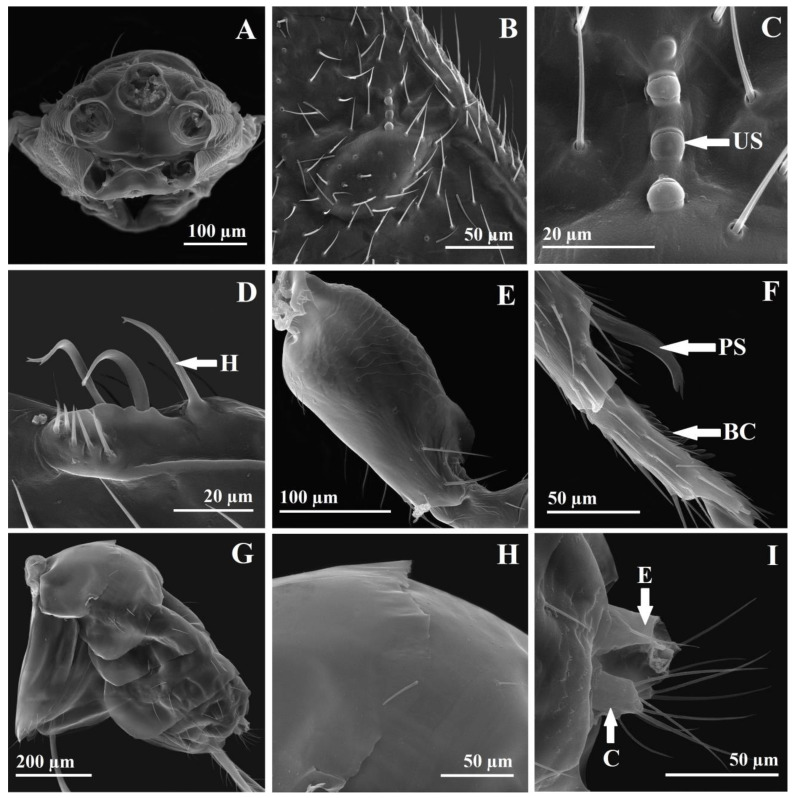
*M. irinae* (♀)—SEM micrographs. (**A**) Metasternum (metasternal area); (**B**) forewing stigma; (**C**) uncus with uncal sensilla (US); (**D**) hindwing coupling structure with hamuli (H); (**E**) metacoxa; (**F**) protibial spur (PS), basitarsal comb (BC); (**G**) metasoma—lateral view; (**H**) metasoma detail; (**I**) epipygium (E), cerci (C).

Relative measurements of mesosoma: ML 65, MW 37, PCL 22, PCW 32, MLL 23, MLW 25, SL 22, SW 24, FL 7, PL 10, PW 28. The mesosoma is 1.75× as long as broad. The pronotal collar is transverse, 0.68× as long as broad, 0.95× as long as the middle lobe of the mesoscutum, and 0.48× as long as the length of the middle lobe of the mesoscutum and scutellum. The middle lobe of the mesoscutum is 0.92× as long as broad and 1.04× as long as the scutellum. The scutellum is 0.91× as long as broad. The frenum is 0.31× as long as the scutellum. The propodeum is 0.35× as long as broad. The pronotal collar is transversal-reticulate-imbricate with three dorsal transversal and one lateral longitudinal line of hairlike setae (Figure 2C,D and Figure 5E,F). The lateral panel of the pronotum is coriaceous-imbricate superiorly and alutaceous-imbricate inferiorly (Figure 5E). The mesoscutum is transversal-reticulate-imbricate anteriorly to alutaceous-imbricate on lateral-anterior face of the lateral lobe of the mesoscutum (Figure 5E,F). On a stereomicroscope, the pronotal collar and anterior part of the middle lobe of the mesoscutum, depending on light, can appear to have cross striation (Figure 2D). The middle lobe of the mesoscutum has 12 long hairlike setae: 4 anterior to the transversal line, 2 central, and 2 lateral, just before the suture with the pronotum, 6 (3 on every side) oblique from the middle side of the middle lobe, from the notauli to the central-posterior part of the middle lobe, and 2 setae posterior lateral (Figure 2D). The lateral lobe of the mesoscutum laterally has four long, hairlike setae (Figure 2C,D). The notauli are complete. The scutellum and axilla are coriaceous-imbricate (Figure 5F,G). The lateral panel of the axilla are alutaceous-imbricate (Figure 5E). The frenal line is difficult to see on a stereomicroscope. The frenum is coriaceous to reticulate (Figure 5G). The scutellum has six long, hairlike setae, three in each of two lateral longitudinal rows (Figure 2D and Figure 5G). The metanotum has a smooth dorsellum and lateral panels, with depressions with three strong longitudinal carinas in front of lateral panels and one small central carina posterior to the dorsellum and three lateral small carinas (Figure 5H). The propodeum is bare, reticulate in the central area, with half of the median carina and 2–3 short, lateral, basal carinas (Figure 5H,I). The distal part has a small transversal smooth collar preceded by three short lateral longitudinal carinas. The postspiracular sulcus is deep, wide, long, reaching the end of the propodeum, smooth inside, with 3–4 thin transverse carinas (Figure 5H). The callus is alutaceous-imbricate with dense, short, thin setae. The prepectus is smooth. The tegula is smooth, basal, pale, alutaceous with setae. The acropleuron is bare, longitudinally striate. The mesepisternum is bare and reticulate, the mesepimeron is bare, transversally striate. The metapleuron is vertically striate (Figure 5E). The propleuron, prosternum, mesosternum are smooth with few small setae. The metasternum (metasternal area) is smooth and bare. The length between the two hind coxal foramen is 9× as long as the distance between the hind coxal foramen and the propodeal foramen (Figure 6A). The diameter of the propodeal foramen is 1.14× the diameter of the hind coxal foramen. Relative length/breadth (ratio) of forewing: 112/47 (2.38). Relative length of forewing veins: SMV 43, PST 15, MV 23, PMV 20, STV 11, STG 10, UNC 3. Relative ratios of forewing veins: SMV/MV 1.86, MV/PMV 1.15, PMV/STV 1.81, STG/UNC 3.33. The stigma is oval-rhombic with a length/breadth (ratio) of 10/5 (2) (Figure 3D, and Figure 6B). The uncus has four uncal sensilla in a row, the last one smaller (Figure 6B,C). The costal cell length/breadth (ratio) is 41/4 (10.25), with three lines of setae on the upper half. The submarginal vein has 11 long setae. The basal cell and speculum are almost bare, with two very small setae (Figure 3D). The basal setal line has three medium-long setae, a cubital setal line (cubital vein) with four long setae basally, well delimited. The marginal setae (fringe) are well developed on the basal upper half of the wing and beyond the postmarginal vein on the superior part, to the apex. Relative length/breadth (ratio) of hindwing: 82/17 (4.82). The hindwing coupling structure has three strong hamuli-like hooks with terminal ramifications and five strong basal bristles (Figure 6D). All coxae, femurs, and tibias are alutaceous-imbricate. The distal part of the fore coxae has wrinkles. Relative length of foreleg: coxa 20, trochanter 7, femur 25, tibia 20, tibial spur 6, tars 30, basitarsus 9. Relative ratios of foreleg: tibia/femur 0.8, tibial spur/tibia 0.3, tibial spur/basitarsus 0.66. The protibial spur has a curved, bifid terminal and is stout (Figure 6F). The basitarsal comb is longitudinal and well developed. Relative length of hindleg: coxa 22, trochanter 7, femur 42/12 (3.5), tibia 33, longer spur 5, shorter spur 2, tars 30, basitarsus 10. Relative ratios of hindleg: tibia/femur 0.78, shorter spur/longer spur 0.4, metatibial longer spur/tibia 0.15, metatibial longer spur/basitarsus 0.5.

**Figure 7:**
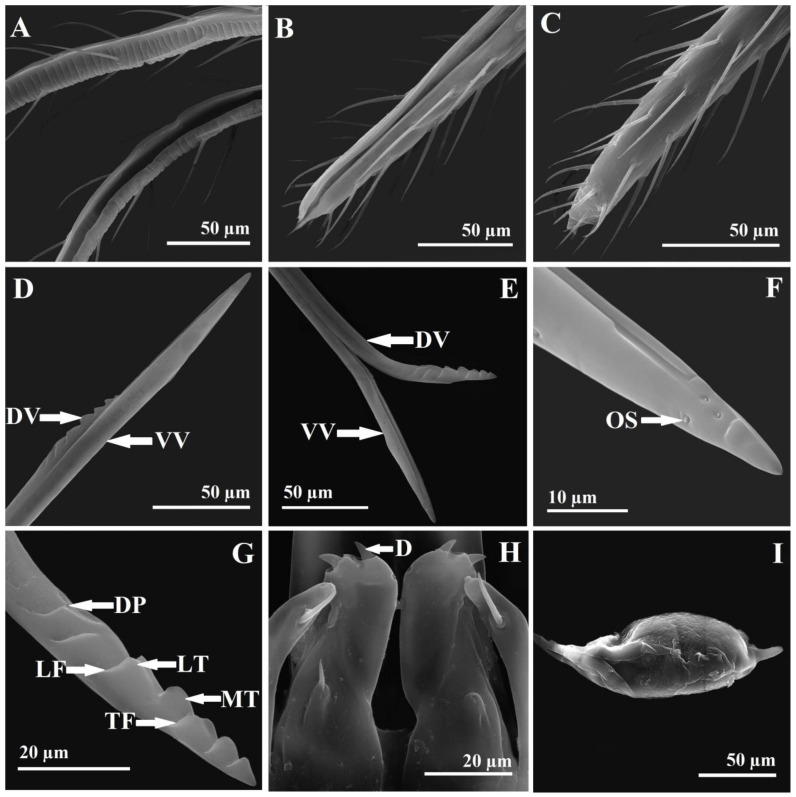
*M. irinae* (♀♂)—SEM micrographs. (**A**) Ovipositor sheaths (third valvulae); (**B**) ovipositor sheaths—internal view; (**C**) ovipositor sheaths—external view; (**D**) ovipositor stylet—ventral valve (second valvulae) (VV) and dorsal valve (first valvulae) (DV); (**E**) ventral valve (VV) and dorsal valve (DV) opened terminal; (**F**) ventral valve with ovipositor (campaniform) sensilla (OS); (**G**) dorsal valve with median tooth (MT), lateral teeth (LT), dorsal plate (DP), transverse furrows (TF), lateral furrows (LF); (**H**) ♂ genitalia with digitus with two dents (D); (**I**) ovarian egg.

Relative length/breadth (ratio) of metasoma: 63/25 (2.52). Relative measurements of body length: body 145, head 25, mesosoma 65, metasoma 63, ovipositor 100. Because of the convexity of the mesosoma and the position of the head in a dead specimen, the length of the body is not equivalent to the addition of the head, mesosoma, and metasoma length, 145 versus 25 + 65 + 63 = 153, the small difference being justifiable. Moreover, because of the sloping position of the first gastral tergite and the convexity of the gaster, the length of the mesosoma is not equivalent to the addition of the gastral tergites lengths. Relative length ratios of body parts: metasoma/mesosoma 0.96, ovipositor/metasoma 1.58, ovipositor/metatibia (ovipositor index) 3.03. The gaster is sessile with a petiole (metasomal tergite 1 is very short) (Figure 2A–D). The posterior margin of gastral tergites is complete, slightly sinuous, without incisions. Relative length of gastral tergites: Gt1 20, Gt2 20, Gt3 15, Gt4 10, Gt5 5, Gt6 3, Gt7 retracted and not possible to measure, Gt8 (syntergum, epipygium) 4, cerci 2. The surface of the abdomen is smooth, bare, with one transversal subterminal to the median line of long, hairlike setae on gastral tergites 1–6 and few scattered, long setae on the distal part of the gastral sternum segments (Figure 6G,H). The syntergum is rounded dorsally, the cerci have five setae, one short basal one and four longer ones, two on the apex and two on the upper part, with the length of the long setae being 2.5× the length of the cerci (Figure 6I). The ovipositor sheaths (third valvulae) have transversal grooves and long setae (Figure 7A–C). The length of setae is 2.5–3× the width of the sheath. The ventral valve (second valvulae) is smooth, with terminal and subterminal ovipositor (campaniform) sensilla (Figure 7D–F). The dorsal valve (first valvulae) is smooth, with three terminal median teeth (the third one is the biggest), two pairs of lateral teeth, and one dorsal plate with a rugose sculpture (Figure 7D,E,G). The tooth area has five transverse furrows and a dorsal plate area with two lateral furrows (Figure 7G). We found six ovarian eggs per female. The ovarian egg is oval with a very long anterior peduncle and a short process at the posterior end (Figure 3J). The length of the anterior peduncle is 6× and the length of the posterior process is 0.16× the length of the egg. The egg surface is smooth (the exochorion is smooth, the endochorion is rugose with imbricate irregular plates and pores) (Figure 7I). The micropyle was not observed.

#### 3.2.2. Allotype ♂

The body length is 1.84 mm. The general aspect of the male is similar to that of the female but with some differences (Figure 2G–I). The body is blacker on the posterior part of the body, especially on the gaster. The propodeum is intensely black in the central area but with a yellow spot in the middle and a black stripe in the upper lateral part, in the upper part of the spiracles. The axilla has a thin black basal line. The stigma is evidently bigger that in the female, rounded, and brown (Figure 3E). The gaster is dorsally intensely amber to amber orange on Gt4 and Gt6, and has intense black stripes dorsally extended laterally on Gt2 and Gt4. Gt1 is intensely black dorsally in the first half and brown on the terminal half; Gt2 is amber dorsally, yellow laterally, with an intense black stripe in the dorsal basal are extended laterally, and one black spot on the terminal median; Gt3 is amber, without black; Gt4 is amber with a black stripe basally and another one, wider, terminally, extended laterally; Gt5 and Gt6 are intensely amber to orange. Relative measurements of the head: HW 45, HH 35, BOF 14, LMS 10, HE 22, DT 4, DAT 3, LATE 9, LTOF 15, HL 29, LT 5, LE 19, IOD 30, OOL 4, POL 10, LOL 4, DMO 5, DLO 5. The head is slightly transverse, 1.28× as wide as high (1.2× for the female) and 1.55× as wide as long (1.6× for the female), the head being a little elongated compared with the female head, and the position of the toruli is a little higher on the face, but no other significant differences compared with the female. Relative length/breadth (ratio) of antennomeres (Figure 3B): flagellum 43; funicle 29; scape 13/4 (3.25); pedicel 7/3 (2.33); anellus 1.8/2.1 (0.85); funiculars: F1 5/3 (1.66), F2 4/4 (1), F3 4/4 (1), F4 4/4 (1), F5 4/4 (1), F6 4/4 (1), F7 4/4 (1); clava: 11/6 (1.8). Compared with the female, the male has a broader clava and a shorter F1–F3 and all the funiculars are quadrate; in the female F1–F3 are a little more elongated than broader. Relative measurements of mesosoma: ML 80, MW 40, PCL 25, PCW 30, MLL 25, MLW 23, SL 25, SW 23, FL 6, PL 13, PW 30. The mesosoma is 2× as long as broad. The pronotal collar is transverse, 0.83× as long as broad, 1× as long as the middle lobe of the mesoscutum and 0.54× as long as the length of the middle lobe of the mesoscutum and scutellum. The middle lobe of the mesoscutum is 1.08× as long as broad and 1× as long as the scutellum. The scutellum is 1.08× as long as broad. The frenum is 0.24× as long as the scutellum. The propodeum is 0.43× as long as broad. The mesosoma is more elongate that in the female, 2× as long as broad, 1.75 in the female. The pronotal collar is more elongated that in the female, 0.83× as long as broad, 0.68× in the female. The scutellum is wider that in the female, 1.08× as long as broad, 0.91 in the female. Relative length/breadth (ratio) of metasoma: 65/30 (2.16). Relative measurements of body length: body 170, head 29, mesosoma 80, metasoma 65. The mesosoma is 0.81× as long as the metasoma, being shorter that in the female (0.96×). Relative length/breadth (ratio) of forewing: 104/45 (2.31). Relative length of forewing veins (Figure 3E): SMV 40, PST 15, MV 21, PMV 20, STV 11, STG 9, UNC 3. Relative ratios of forewing veins: SMV/MV 1.90, MV/PMV 1.05, PMV/STV 1.81, STG/UNC 3. Stigma round with length/breadth (ratio): 9/7 (1.28). Costal cell length/breadth (ratio): 40/4 (10). The main difference between the forewings of the male and female specimens is bigger and rounded stigma on the male and more setae on the basal cell and speculum. Relative length/breadth (ratio) of male genitalia (Figure 3I): 32/10 (3.2). Relative length of male genitalia parts: phallobase 20, paramere 5, volsella 10, digitus 3, aedeagus 12. The aedeagus is 0.6× as long as the phallobase. The digitus has two dents (Figure 7H). The volsella has one sensilla trichodea in the middle and a paramere with two sensilla trichodea terminally.

#### 3.2.3. Variation

The females’ lengths were around 1.7 mm, with 1.3 mm being the smallest and a little more than 2 mm the biggest. We chose a relatively normal to smaller female to be the holotype because it was the only one with large, open mandibles and it was easy to see the mandible dents and the bilobed clypeus, characteristics more difficult to see with closed mandibles, and the operation to open the mandibles can destroy the specimen. The male allotype measured 1.84 mm and was a very big specimen; we especially chose it to be the allotype as it is easier to see characteristics on a big specimen. The males’ lengths were around 1.5–1.6 mm, with 1.18 mm the smallest and 1.84 the biggest (allotype). In smaller male specimens, the gaster is complete with an intense amber color, blacker dorsally and the propodeum without black or with a thin black stripe anteriorly, and the axilla with blacker basally.

### 3.3. Sex Ratio

104♀♀/24♂♂ (4.33).

### 3.4. Host Plant

*Megastigmus irinae* was obtained from seeds of *Pemphis acidula* J. R. Forst & G. Forst (Lythraceae).

### 3.5. Distribution

Maldives: Lhohifushi Island, North Male Atoll; Kihaadhuffaru Island, Southern Maalhosmadulu Atoll from Baa Atoll.

### 3.6. Etymology

The species is named after Dr. Gostin Irina Neta, a Romanian plant histology professor and researcher, in recognition of her passion for the Maldives and her significant contribution to planning the expeditions in the Maldives Archipelago, which made the discovery of this new species possible.

### 3.7. Diagnosis

*Megastigmus irinae* generally can be distinguished from other related species of *Megastigmus* by a combination of the following characteristics: a small dimension, a length around 1.6–1.7 mm, a yellow body with scattered black setae, a dorsal mesosoma reticulate-imbricate, a scutellum with six long, hairlike setae, a frenum coriaceous to reticulate, a propodeum reticulate in the central area, with half of the median carina, a male digitus with two dents, a female ovipositor with a dorsal valve with three median teeth (third one the biggest) and two pairs of lateral teeth, a tooth area with five transverse furrows, and a dorsal plate area with two lateral furrows, and the host plant (seeds of *Pemphis acidula*).

## 4. Discussions

*Megastigmus irinae* corresponds to the diagnoses for the Megastigmidae family [6,26]: antenna with 10 flagellomeres (Figure 2E and Figure 3A), a bilobed clypeus (Figure 4B), mandibles with three teeth (Figure 2F and Figure 5A), occipital carina present (Figure 4G,H), an elongated pronotum (Figure 2A–D and Figure 5E,F), complete notauli (Figure 2D and Figure 5E,F), a frenum with a distinct frenal groove (Figure 2D and Figure 5G), a postmarginal vein longer than the stigmal vein (Figure 3D), legs with five tarsomeres (Figure 3G,H), a relatively short metacoxa (Figure 2A–C and Figure 3H), a stout and curved protibial spur (Figure 3G and Figure 6F), a longitudinal basitarsal comb (Figure 6F), a metasomal apex with a separate epipygium in females (Figure 6I), elongated cerci (Figure 6I), long and upcurved ovipositor sheaths (Figure 2A,B). This is the first mention of the family Megastigmidae in the Maldives.

In the key to genera of Australasian Megastigmidae [9] (Megastigminae at that time) we easily go with *M. irinae* to the *Megastigmus* genus. This is the first mention of the genus *Megastigmus* in the Maldives.

In the key for Indian species of *Megastigmus* [27], *M. irinae* goes to the first paragraph of couplet 10, with the POL as long as the OOL as in *M. albizziae* Mukerji, but it differs with an SMV 1.86× as long as the MV (7× in *M. albizziae*), the STV 0.47× as long as the MV (the STV is as long as the MV in *M. albizziae*), the clava 3× as long as F7 (1.7× in *M. albizziae*), the scape 2.33× as long as the pedicel (3.75 in *M. albizziae*) (Figure 3A,D). Moreover, the hosts differ, *M. albizziae* being obtained from pods of *Albizzia* and *M. irinae* from seeds of *Pemphis acidula* (Figure 1D–N). If we do not consider the POL as long as the OOL in *M. irinae* and try to go to couplet 11 with the POL 2.3–2.5× as long as the OOL, there are also other differences: the SMV is 1.86× as long as the MV versus 1.92–2.33×, the STV 0.47× is as long as the MV versus 0.44–0.52×, the clava is 3× as long as F7 versus 2.3–2.6×, the scape 2.33× is as long as the pedicel, versus 2.17–2.6×. Just the last characteristic can be shared, but especially the POL as long as the OOL versus the POL 2.3–2.5× as long as the OOL and the clava 3× as long as F7 versus 2.3–2.6× stop us from going further. *M. irinae* also differs from *M. albizziae* by other characteristics. On *M. albizziae,* the general body color is yellowish brown versus yellow on *M. irinae* (Figure 2A–I), F1 is shorter than F2 versus F1 being 1.25× as long as F2 (Figure 3A), the mesoscutum has fine transverse striations versus being reticulate-imbricate (Figure 5E,F), the propodeum is without a median carina versus one half of a median carina (Figure 5H,I), and the dimensions, 1.7 versus 3–4 mm, are even bigger in the male specimen [28]. The two species have in common the reticulate scutellum and propodeum. The distribution of *M. albizziae* is restricted to India (Dehli and Tamil Nandu state from India) [28,29].

In the key of the females of *Megastigmus* for Eastern and Southern Africa [30], *M. irinae* goes to couplet 5, and we have to choose between minute species with a body length less than 1.5 mm, a pale pilosity on the mesosoma, and two pairs of hairs on the scutellum and larger species with a body length more than 2.5 mm, a dark pilosity on the mesosoma and 3–8 pairs of hairs on the scutellum. The females of *M. irinae* have lengths around 1.7 mm, a dark pilosity on the mesosoma and three pairs of hairs on the scutellum (Figure 2D and Figure 5G). If we choose to go to the first paragraph, we go to couplet 6 and we must choose between a body color mostly black and a body color nearly completely orange yellow. *M. irinae* is almost completely yellow (Figure 2A–I), so we go close to *M. icipeensis* Roques & Copeland. *M. irinae* differs from *M. icipeensis* in dimension, 1.7 versus 1.2 mm, a yellow body versus orange-yellow, a dark pilosity on the mesosoma versus a pale pilosity on the mesosoma, three pairs of hairs on the scutellum versus two (Figure 2D and Figure 5G). It is interesting that in Figures 62 and 64 from Roques et al. [30], we can clearly see three hairs on the left side of the scutellum and two hairs on the right side. *M. irinae* also differs from *M. icipeensis* with a smaller, oval-rhombic stigma with a length/breadth ratio of 2 (Figure 3D) versus a bigger, oval stigma with length/breadth ratio of 1.4, a head 1.65× as wide as long versus 1.2×, a POL 2× as long as the OOL versus 2.7×, a dorsal mesosoma reticulate-imbricate (Figure 5E–I) versus a smooth one with fine transverse striae, a middle lobe of the mesoscutum 1.04× as long as the scutellum versus 0.9, a scutellum 0.91× as long as broad versus 1.1, a reticulate propodeum with a half of a median carina (Figure 5H,I) versus a smooth one with a zig-zag carina, an MV/PMV of 1.15 versus 0.8. If we choose to go to the second paragraph of couplet 5, we arrive at *M. transvaalensis* (Hussey), but *M. irinae* has a stigmal vein 1.1× as long as the length of the stigma versus 0.4×, a noninfuscate versus infuscate stigma, a cubital setal line (cubital vein) with four long basal setae (Figure 3D) versus a bare basal cubital setal line bare, three pairs of hairs on the scutellum (Figure 2D and Figure 5G) versus four, an ovipositor 1.58× as long as the metasoma versus 1.4, a basal setal line almost hyaline in males of *M. irinae* (Figure 3E) versus strongly brown in males of *M. transvaalensis*, a more elongated mesosoma on *M. transvaalensis*, a middle lobe of the mesoscutum 0.92× as long as broad versus 1.5×, a yellow, reticulate propodeum with a half of a median carina (Figure 5H,I) versus one black in the middle, without a median carina but with oblique carinae [22,30], a pronotum and a middle lobe of the mesoscutum that are reticulate-imbricate (Figure 5E,F) versus with cross-striations, a male genitalia digitus with two dents (Figure 3I and Figure 7H) versus three [22,30], females 1.7 mm in length versus 2–3 mm. Males of *M. irinae* do not exhibit as much color variation as males of *M. transvaalensis*, where small specimens are yellow with a little infuscate stigmal area, intermediate specimens are yellow with some black and a more infuscate stigmal area, and the largest specimens are black with some yellow and an extensive infuscate stigmal area. *M. transvaalensis* has as host plants, species from Anacardiaceae (*Schinus* spp., *Rhus* spp.) [22,30,31,32].

For Japan, Kamijo [33] divided *Megastigmus* species in two groups, entomophagous and phytophagous, in fact, the actual *Bootanomyia* and *Megastigmus* genera. In the key for phytophagous species, *M. irinae* goes to couplet 3, where we must choose between a frenum longitudinally sculptured as is in *M. aculeatus* (Swederus) and a frenum nearly smooth to go to couplet 4. In fact, the frenum of *M. irinae* cannot go to any of these two options, being coriaceous to reticulate (Figure 5G,H). *M. irinae* is completely distinct from the common Palearctic species *M. aculeatus*. The latter is larger, measuring over 3 mm in length, and exhibits a more extensive black on the body, a black antenna, a black frons around ocelli, a temple with a big black area, a completely black anterior part of the mesoscutum and propodeum, black metacoxa and axilla, extensive black dorsally on the gaster [34]. *M. irinae,* in comparation, is almost completely yellow (Figure 2A–I). Additionally, *M. aculeatus* exhibits strong cross-striations on the entire dorsal mesosoma, while *M. irinae* displays a reticulate-imbricate pattern (Figure 5E–I). The propodeum in *M. aculeatus* has two median longitudinal carinas with two big lateral depressions; on *M. irinae,* the propodeum is reticulate in the central area, with half of a median carina (Figure 5H,I). On the ovipositor of *M. irinae,* the dorsal valve (first valvulae) has three median teeth (the third one is the biggest) and two pairs of lateral teeth (Figure 7D,E,G); on *M. aculeatus,* there are one median tooth terminal and three pairs of lateral teeth [22]. On the male genitalia of *M. irinae,* the digitus has two dents (Figure 3I and Figure 7H) versus three on *M. aculeatus* [22]. *M. aculeatus* has as a host plant seeds of *Rosa* spp. [22].

In the West Palearctic key for native and introduced species of *Megastigmus* [22], *M. irinae* goes to couplet 11, and we have to choose between a testaceous dorsal mesosoma with a frenum with strong longitudinal carinae and a dorsal mesosoma with some brown or black areas and a smooth frenum. With a yellow dorsal mesosoma (Figure 2A–I) and a reticulate-imbricate frenum (Figure 5G,H), *M. irinae* cannot go further in the key. Even if we force the key to go further to both paragraphs of couplet 11, we cannot find appropriate characters for *M. irinae* to follow the key.

This is the first mention of a species from the Megastigmidae family having as a host plant a species from the Lythraceae family. It is also the first mention of an association of a species from Chalcidoidea (Hymenoptera) with the genus *Pemphis* [1]. We obtained in laboratory conditions 3 ♀♀ and 6 ♂♂ of *M. irinae* from 91 capsules (fruits) of *Pemphis acidula* (Figure 1D–K) collected on 25 July 2018 from Lhohifushi Island (Figure 1B) and 25 ♀♀ and 12 ♂♂ of *M. irinae* from 161 capsules of *Pemphis acidula* collected on 20 September 2022 from Kihaadhuffaru Island (Figure 1C). The fact that we obtained adults of *M. irinae* from capsules of *P. acidula* collected on 25 July 2018 and on 20 September 2022 led us to the supposition that because of the tropical climate with very homogenous conditions throughout the year, the larvae developed in the seeds throughout the year, and not just at certain times of the year as in a temperate climate on other species of *Megastigmus*. At first look, we obtained cumulatively 28 ♀♀ and 18 ♂♂ of *M. irinae* from 252 capsules of *P. acidula*. In both cases, the adults of *M. irinae* emerged from capsules of *P. acidula* in the next days from the collecting date but no more than one month later. We found 45 capsules with emerging holes, 44 capsules with one emerging hole (Figure 1G–K), and 1 capsule with two emerging holes (Figure 1I). In almost all cases, we found one specimen of *M. irinae* emerging from one capsule of *P. acidula*. In only one case, we found two specimens of *M. irinae* emerging from one capsule of *P. acidula*. One specimen of *M. irinae* develops in one seed of *P. acidula* (Figure 1L–N). We found only six ovarian eggs in one female, and this can be an explanation for the fact that a female puts just one egg in one capsule of *P. acidula*, even though the capsule has many seeds. This increases the chance of the survival of the larvae in case of the destruction of the capsule, many larvae in many capsules being a better choice. Sometimes, when emerging from the seed, the adult of *M. irinae* can also damage 1–2 adjacent seeds to the one serving as food for the larvae. In Figure 1L, we can observe the first seed, located at the top, which has an emergence hole for the adult of *M. irinae*. Additionally, there is another seed at the bottom that shows a slight damage caused by the adult of *M. irinae* as it exits the capsule of *P. acidula*. The percentage of capsules of *P. acidula* infested by *M. irinae* was 0.08% for Lhohifushi Island and 0.22% for Kihaadhuffaru Island. *M. irinae* is a strictly phytophagous species, one larva consuming a single seed inside.

The adults of *M. irinae* can be collected on *P. acidula* plants, as they are observed flying around and walking on the plants. We collected 76 ♀♀ and 6 ♂♂ by sweeping with an entomological net on plants of *Pemphis acidula*, 53 ♀♀ and 6 ♂♂ on Lhohifushi Island, and 23 ♀♀ on Kihaadhuffaru Island. With a good eye and patience, it is even possible to observe the adults of *M. irinae* on the plants of *P. acidula*. *M. irinae* shows a preference for isolated plants near the seaside, in the coastal areas close to the water, and in open spaces with abundant sunlight (Figure 1D). They are not commonly found on plants located far from the water, in the interior of the islands, or in areas with dense vegetation and shaded spaces. We did not collect a single specimen using any other method apart from using an entomological net on the plants of *P. acidula* and rearing from the capsules of the host. We used yellow traps and extensive sweeping with the entomological net on the very scarce herbaceous vegetation from the islands and on other plants, but we did not capture a single exemplar of *M. irinae*. We conclude that the adults of *M. irinae* stay just on the plants of *P. acidula*.

The Maldives is the lowest country in the world, with a natural ground level of 1.5 m on average and more than 80% of the islands being less than one meter above sea level. The Maldives Archipelago is at high risk of being submerged due to rising sea levels because of global climate changes [35]. A few dozen islands are now under the sea level and disappearing. Thus, the future of the tiny *M. irinae* can also depend on the global human activity. The local human activity can also affect *M. irinae*, especially by affecting the host plant, particularly on inhabited islands, where the impact of the human population on the local biodiversity can be extreme because of the concentrations of many people on very small islands. On tourist resort islands, the situations are more complex. Some resort islands have little vegetation, others have few plants, but there are resorts with a lot of plants. Resorts can be established on almost bare islands and can be populated with plants by humans and, in other cases, a resort can be established on an uninhabited island with a lot of vegetation, as the recent case of Furaveri Island. Some islands are used as touristic picnic islands, others for agricultural and industrial purposes, a few for garbage, and even as a cemetery islands. Therefore, the uninhabited islands are in permanent danger of being used by humans for tourism or for other activities. One big impact on insects from the resort islands can be the fumigations against mosquitos, two times per day. Fortunately, *M. irinae* prefer the plants of *P. acidula* from the seaside where the impact of fumigation is the lowest.

## 5. Conclusions

At least for the moment, *Megastigmus irinae* is an endemic species for the Maldives Archipelago, found in Lhohifushi and Kihaadhuffaru islands (Figure 1A–C). The expeditions in Nalaguraidhoo, Dhigurah, Maamigili, Dhiffushi, Furaveri, Iru Veli, and Hulhumale islands (Figure 1A) have no results yet. However, if we look at the distribution of the host plant, *P. acidula*, from East Africa to India, Southeast Asia, Japan, Australia, Micronesia, the Pacific Islands, and French Polynesia, we expect that future research will extend considerably the known area of *M. irinae*. Böhmová et al. [8] finished their paper with “apart from a few *Megastigmus* species with economic importance, most other Megastigmidae are rather poorly studied in terms of taxonomy but also of biology” and Bouček wrote in his monumental *Australasian Chalcidoidea* [9] about *Megastigmus* genus that a “careful examination of every particular case is much needed”. We conclude that this research makes a little light on the Megastigmidae family and *Megastigmus* genus by discovering a surprising new species with interesting data on its morphology and biology, from the paradise of the Maldives Archipelago, with some perspectives on the islands’ biodiversity and biogeography, climate change and habitat destruction.

## Data Availability

The data generated in this study are provided here and they are also available upon request.
